# Distinct and shared patterns of brain plasticity during electroconvulsive therapy and treatment as usual in depression: an observational multimodal MRI-study

**DOI:** 10.1038/s41398-022-02304-2

**Published:** 2023-01-10

**Authors:** Tobias Bracht, Sebastian Walther, Sigrid Breit, Nicolas Mertse, Andrea Federspiel, Agnes Meyer, Leila M. Soravia, Roland Wiest, Niklaus Denier

**Affiliations:** 1 Translational Research Center, University Hospital of Psychiatry and Psychotherapy, University of Bern, Bern, Switzerland; 2 Translational Imaging Center (TIC), Swiss Institute for Translational and Entrepreneurial Medicine, Bern, Switzerland; 3 Institute of Diagnostic and Interventional Neuroradiology, University of Bern, Bern, Switzerland

**Keywords:** Molecular neuroscience, Depression

## Abstract

Electroconvulsive therapy (ECT) is a highly effective treatment for depression. Previous studies point to ECT-induced volume increase in the hippocampi and amygdalae, and to increase in cortical thickness. However, it is unclear if these neuroplastic changes are associated with treatment response. This observational study aimed to address this research question by comparing neuroplasticity between patients with depression receiving ECT and patients with depression that respond to treatment as usual (TAU-responders). Twenty ECT-patients (16 major depressive disorder (MDD), 4 depressed bipolar disorder), 20 TAU-responders (20 MDD) and 20 healthy controls (HC) were scanned twice with multimodal magnetic resonance imaging (structure: MP2RAGE; perfusion: arterial spin labeling). ECT-patients were scanned before and after an ECT-index series (ECT-group). TAU-responders were scanned during a depressive episode and following remission or treatment response. Volumes and cerebral blood flow (CBF) of the hippocampi and amygdalae, and global mean cortical thickness were compared between groups. There was a significant group × time interaction for hippocampal and amygdalar volumes, CBF in the hippocampi and global mean cortical thickness. Hippocampal and amygdalar enlargements and CBF increase in the hippocampi were observed in the ECT-group but neither in TAU-responders nor in HC. Increase in global mean cortical thickness was observed in the ECT-group and in TAU-responders but not in HC. The co-occurrence of increase in global mean cortical thickness in both TAU-responders and in ECT-patients may point to a shared mechanism of antidepressant response. This was not the case for subcortical volume and CBF increase.

## Introduction

Electroconvulsive therapy (ECT) is a highly efficient therapy for the treatment of depression [1, 2]. Response rates range from about 58% for patients with treatment resistant depression (TRD) to 70% for patients with a depressive episode without a history of failure to respond to medication [3]. Consequently, the clinical utility of ECT for the treatment of depression has fostered a plethora of research on its neurobiological underpinnings [4–9].

One of the most replicated findings is an ECT-induced volume increase in the hippocampi and the amygdalae [7, 10, 11], which may reverse depression pathophysiology linked to these limbic structures [12–14] and to extended networks [15–17]. However, studies investigating correlations between hippocampal and amygdalar enlargements during ECT and clinical improvements led to conflicting results [18–21]. A recent meta-analysis suggests that overall volume increase in the hippocampi is not associated with response [7], which is in line with findings of a large mega-analysis [4]. Thus, overall enlargements of the hippocampi may rather be an unspecific effect of ECT than the driving force for treatment response.

Another explanation for heterogeneity in findings is the assumption that neuroplasticity in sub-compartments of the hippocampi underlie treatment response in depression [7]. These subtle changes may not be detected if investigating the entire hippocampus. While some studies found that volume increase in the dentate gyrus is associated with treatment response [22], a recent meta-regression analysis revealed the opposite association [7]. Furthermore, volume enlargement in the anterior but not in the posterior hippocampus was associated with treatment response [23]. In contrast, responders had an increase in cerebral blood flow (CBF) in the right middle and left posterior but not in the anterior hippocampus [5, 23]. Therefore, it was suggested that anterior and posterior hippocampi may be linked to distinct functional networks related to treatment response [23]. In addition, some groups linked ECT-treatment response to increase in cortical thickness in regions including the insula, the anterior cingulate cortex (ACC), and the orbitofrontal cortex (OFC) [19, 24, 25]. However, others failed to replicate such associations [20, 26].

We aim to investigate which ECT-induced changes are of clinical relevance for treatment response in depression. We assume that neuroplastic changes that are relevant for treatment response should resemble plasticity in patients with depression that respond to other treatments. Indeed, similar pattern were observed: One longitudinal study found that successful treatment with antidepressive medication prevents the hippocampus from progressive atrophy [27]. Others found hippocampal volume increase in remitters and responders to antidepressant medication [28, 29]. Furthermore, increase in cortical thickness was found in remitters to treatment as usual (TAU) in the anterior cingulate cortex (ACC) and in the orbitofrontal cortex (OFC) [28, 30, 31]. However, these findings were not replicated in a large longitudinal study [32]. Moreover, none of these studies compared their findings in patients with longitudinal MRI-assessments in a healthy comparison group, limiting the interpretability of longitudinal changes in disease. To further advance our understanding which ECT-induced neuroplastic changes are responsible for treatment response there is need for longitudinal studies that statistically compare neuroplastic changes of ECT-patients with treatment responders receiving TAU and with HC [33].

This study aimed to compare ECT-induced neuroplasticity with the neurobiological underpinnings of response to TAU. Therefore, multimodal MRI data were acquired twice (baseline and follow up) in: (1) Patients with depression before and after an ECT-index series. (2) Patients with depression receiving TAU during a depressive episode and following remission or treatment response (TAU-responders). (3) Healthy controls (HC). We hypothesized the following concurrent changes in the ECT-group and in TAU-responders: First, volume increase in the hippocampi and the amygdalae. Significant results were followed up with exploratory group comparisons for anterior and the posterior hippocampi, hippocampal subfields and subnuclei of the amygdalae. Second, increase in CBF in the hippocampi and the amygdalae. Third, increase in global mean cortical thickness, and specifically in the insula, ACC and OFC [19, 24, 25]. Fourth, we hypothesized that identified changes are associated with reductions of depression severity.

## Methods

### Participants

Patients of this observational study were recruited at the University Hospital of Psychiatry and Psychotherapy in Bern, Switzerland and includes participants of previous reports of cross-sectional data [14, 17, 34]. We recruited two patient groups with a current depressive episode (ECT-group, and TAU-group) and a HC group (for study flow see Supplementary Material, S1). Both patients with a diagnosis of MDD or bipolar disorder according to the Diagnostic and Statistical Manual of Mental Disorders (DSM-5), American Psychiatric Association [35] were included in the ECT group. The TAU-group consisted entirely of patients with MDD. Sixteen ECT-patients had a diagnosis of MDD; four ECT patients were diagnosed with bipolar disorder. All ECT-patients were treatment resistant to previous trials of antidepressive medication or did not tolerate prescribed medication. Two ECT-patients had a history of a previous ECT-treatment. Due to the naturalistic design of our study patients were not required to be on stable medication to participate in the study and antidepressive medication was modified during study participation (for medication changes see Table 1). All patients (ECT and TAU) received multimodal treatment consisting of medication, structured psychotherapy and case management. Patients that were scheduled to begin an ECT-treatment were asked if they were interested to participate in the study. HC were recruited through postings and leaflets. The HC-group was matched to the ECT-group for age and sex. Sample size was 20 for each group. Age range for all participants was 20–65 years. Exclusion criteria for patients were neurological disorders, addictive disorders, psychotic disorders and personality disorders. Comorbid symptoms of anxiety disorders were allowed due to the high comorbidity with depression. A history of ECT was an additional exclusion criterion for the TAU-group. Exclusion criteria for HC were any psychiatric or neurological disorders. Claustrophobia or any other contraindication to perform an MRI-scan were exclusion criteria for all participants. Written informed consent was obtained from all subjects and the study was approved by the local cantonal ethics committee (KEK-number: 2017-00731).

**Table 1:** Demographics for patients and healthy controls.

	ECT (*n* = 20)		TAU (*n* = 20)		Controls (*n* = 20)		*P* value	
Age (years)	44.9 ± 12		44.2 ± 15		43.6 ± 14		0.96	
Sex *n* (female)	8		14		8		0.09	
Handedness *n* (right, left, ambidextrous)	15, 2, 3		17, 2, 1		17, 2, 1		0.78	
BDI-II (Baseline)	30.6 ± 8.1		26.8 ± 8.8		1.5 ± 2.4		0.17	
HAMD-21 (Baseline)	21.4 ± 5.3		22.6 ± 5.1		0.65 ± 1.0		0.49	
Duration of episode (months)	19.8 ± 17		8.2 ± 8		n.a.		**0.01**	
Number of episodes	5.2 ± 4		2.7 ± 2		n.a.		**0.03**	
Time between scans (days)	52.6 ± 24		67.3 ± 29		61.2 ± 17		0.16	
BDI-II (Follow up)	20.8 ± 10.3		13.1 ± 8.4		1 ± 1.8		**0.02**	
HAMD-21 (Follow up)	10.9 ± 8.1		5.6 ± 3.2		0.25 ± 0.8		**0.01**	
*n* (remitter^1^)	9		15		n.a.		0.053	
*n* (responder or remitter^2^)	11		20		n.a.		**0.001**	
*n* (non-responder)	9		0		n.a.		**0.001**	
Medication	T1	T2	T1	T2	T1	T2	T1	T2
SSRI (%)	20	15	25	25	0	0	0.71	0.43
Dual antidepressants (%)	55	50	35	40	0	0	0.20	0.53
Tricyclic antidepressants (%)	15	20	25	30	0	0	0.43	0.47
Tranylcypromine (%)	5	10	0	0	0	0	0.31	0.15
Moclobemide (%)	5	5	0	0	0	0	0.31	0.31
No antidepressant (%)	10	5	20	5	100	100	0.38	0.39
Lithium (%)	35	30	15	20	0	0	0.14	0.47

Age, sex and handedness were compared between ECT, TAU-responders, and healthy controls using an ANOVA and χ^2^ tests. Clinical variables were compared between ECT and TAU-responders using two-sample *t*-tests or Chi-Square tests. Antidepressive medication was compared at T1 (day of the 1st MRI-scan) and at T2 (day of the 2nd MRI-scan).

*ECT* electroconvulsive therapy, *TAU* treatment as usual, *HC* healthy controls.

^1^Remitter: HAMD-21 < 8 points.

^2^Responder: HAMD-21 reduction > 50%.

*P*-values marked with bold indicate statistically significant differences between groups (*p* < 0.05).

The treating psychiatrist made the diagnosis, which was confirmed using clinical case files and the Mini International Neuropsychiatric Interview (MINI) [36]. Comorbid personality disorders were screened using the Structured Clinical Interview for DSM-IV Axis II (SCID-II) [37]. Handedness was assessed with the Edinburgh Handedness Inventory [38]. Depression severity in patients was assessed using the 21-item Hamilton rating scale for depression (HAMD) [39] and the 21-item Beck Depression Inventory-II (BDI-II) [40]. Assessments took place at the days of the MRI-scans. MRI-scans of the ECT-group took place before and after an ECT-index series. The clinical course in the TAU-group was tracked every 4 weeks using the HAMD and the BDI-II. Once remission was achieved (HAMD-total score <8) the second MRI-scan was performed. In case remission was not achieved within 12 weeks after the first MRI-scan the second MRI scan was performed, and TAU-patients were either classified as responders (HAMD-total score reduction ≥50%) or as non-responders (HAMD-total score reduction <50%). In the present study, only remitters and responders (TAU-responders) were included. Sample size of TAU non-responders (*n* = 4) was too small to serve as an additional comparison group (results for TAU non-responders are reported in the supplementary material, S2). HC were screened for psychiatric disorders using the MINI and the SCID-II. HAMD and BDI-II were used to assess subclinical depressive symptoms.

### ECT treatment

ECT-treatment was performed with a Thymatron IV system at the anesthetic recovery room of the University Hospital in Bern (Inselspital), Switzerland. Seventeen patients started with right unilateral (RUL), two patients with bitemporal, and one patient started with bifrontal stimulation. Decision on electrode positioning was made based on the clinical assessment (e.g. severity of depression, knowledge about effective settings of previous ECT-treatments). All patients started with an initial pulse width of 0.5 msec (L0.5-mode). Titration based method was used to determine the initial seizure threshold. Stimulation intensity was five times the initial seizure threshold for RUL stimulation and two times the initial seizure threshold for bitemporal and bifrontal stimulation. During the ECT-index series, five patients were switched from right unilateral to bitemporal stimulation and one patient was switched from right unilateral to bifrontal stimulation. Modifications (e.g. switch of electrode positions, increase of stimulus intensity, and switch to DGX- or 2x-mode) were made depending on the clinical course. The Thymatron IV system automatically adjusts the frequency for any given percent energy setting (stimulus intensity) for any preset program. The range of frequency was 30–70 Hz. ECT-patients had an average of 12.7 ± 4 ECT-sessions between MRI-scans. Etomidate was used for general anesthesia and succinylcholine was used for muscle relaxation. Seizure quality was assessed and monitored using electroencephalogram, electromyography and electrocardiography recordings according to standard clinical routine.

### MRI data acquisition

Each participant was scanned twice with a 3 Tesla MRI scanner (Magnetom Prisma, Siemens, Erlangen, Germany) and a 64-channel head and neck coil at the Swiss Institute for Translational and Entrepreneurial Medicine (SITEM) in Bern. The ECT-group was scanned before and after an ECT-index series. The TAU-group was scanned following study enrolment and after achieving remission or 12 weeks after the first MRI-scan. The HC group was scanned twice with an inter-scan interval similar to the patient groups.

For acquisition of anatomical and high-resolution T1-weighted data, we used a bias-field corrected MP2RAGE sequence for acquisition of two gradient volumes (INV1 and INV2) and computation of a high-contrast volume (UNI). We used the following parameters for acquisition of the MP2RAGE volumes: FOV = 256 × 256, matrix = 256 × 256, slices = 256, voxel resolution = 1 × 1 × 1 mm^3^, TR/TE = 5000/2.98 ms, TI = 700 ms and T2 = 2500 ms.

For the measurement of CBF, we used a pulsed arterial spin labeling (PASL) PICORE Q2TIPS technique [41, 42]. We acquired 90 pairs of label/control volumes in axial direction with a tagging bolus duration (TI1) of 700 ms saturation and an inversion time (TI2) of 2200 ms, whereas during TI2 the labeled blood perfuses the brain tissues causing a decrease of the MR signal. Additional parameter for PASL were FOV = 230 × 230 mm^2^, matrix = 64 × 64, slices = 22, voxel resolution = 3.6 × 3.6 × 6.0 mm^3^, TR/TE = 3300/13 ms, flip angle = 90° and PICORE Q2T perfusion mode. We acquired 181 volumes including a T2-weighted volume at the beginning of the acquisition.

Field map imaging was performed with a double-echo spoiled gradient echo sequence (TR = 500 ms, TE = 4.92/7.38 ms, voxel size: 2.4 × 2.4 × 2.4, flip angle 60°) that generated a magnitude and a phase image (second TE) for computation of the field map.

### MRI pre-processing

#### Structural MRI

**Fig. 1:**
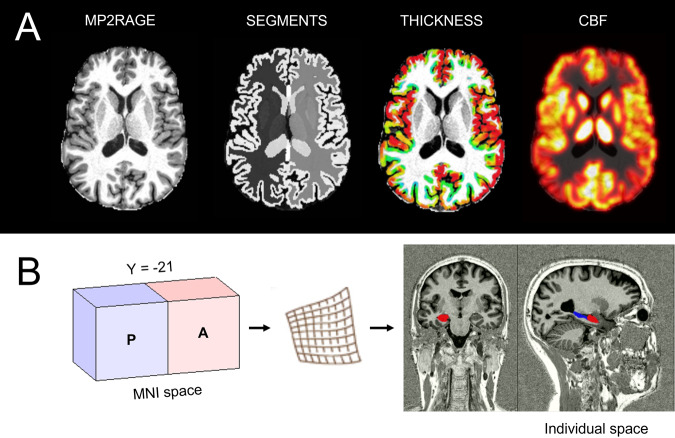
Multimodal MRI measurements. **A** MRI measurements in volume (segments), cortical thickness, and CBF. **B** Segmentation of anterior (**A**) and posterior (**B**) subparts of the hippocampi.

For segmentation and calculation of global mean cortical thickness and subcortical volumes of bilateral hippocampi and amygdalae, we used first *HD-BET* for highly accurate brain extraction using the INV1 volumes [43] and then *DL* + *DiReCT* for further cortical and subcortical segmentation using the brain-masked UNI volumes [44]. *DL* + *DiReCT* applies deep learning-based neuroanatomy segmentation [45] followed by diffeomorphic registration-based cortical thickness estimation (see Fig. 1A). The method was validated with a large cohort and shows similar robustness (test-retest reliability) than *FreeSurfer* [44]. However, *DL* + *DiReCT* was more sensitive than *FreeSurfer* to detect changes in cortical thickness related to age [44], dementia [44] and multiple sclerosis [46] and to identify hippocampal sclerosis in epilepsy [47]. For both hemispheres, *DL* + *DiReCT* was used to extract global mean cortical thickness, and mean thickness of bilateral insulae, caudal ACC and medial and lateral OFC. Automatic subcortical segmentation was based on the AsegAtlas [48]. Resulting volumes of bilateral hippocampi and amygdalae were normalized for total brain volume. For computation of anterior and posterior hippocampi, we used the uncal apex as a standard for landmark-based segmentation of the hippocampi by defining a separation plane in standard MNI space of y = −21 [49]. Using the statistical parametric mapping package (*SPM12*), we inversely transformed binary blocks separated by the separation plane using non-linear transformation to individual space. Finally, we computed intersections of the transformed binary blocks with individual hippocampal segments (see Fig. 1B). Estimates of cortical thickness and subcortical volumes derived from *DL* + *DiReCT* were compared with results derived from *FreeSurfer* (see Supplementary Material, S3) using Pearson correlation coefficients and Cronbach’s alpha (α) demonstrating good to excellent internal consistency for all variables (mean α = 0.90 ± 0.05, range α: 0.81–0.95) (see Supplementary Material, S4).

### Regional CBF

We analysed PASL data using own written MATLAB programs (MATLAB R2021a, MathWorks, USA). First, we realigned raw PASL volumes to reduce movement artefacts. This was followed by a field map correction to all realigned volumes. We quantified CBF time-series using the following relation: ${{{\mathrm{CBF}}}} = {\textstyle{{{\Delta}{{{\mathrm{M}}}}} \over {2{{{\mathrm{M}}}}_0{{{\mathrm{TI}}}}1}}} \cdot {{{\mathrm{e}}}}^{{\textstyle{{{{{\mathrm{TI}}}}2} \over {{{{\mathrm{TIb}}}}}}}},\left[ {{{{\mathrm{CBF}}}}} \right] = {\textstyle{{{{{\mathrm{ml}}}}} \over {100\,{{{\mathrm{g}}}}\,{{{\mathrm{min}}}}}}}$, where ∆M is the difference signal (control – labeling), M_0_ the equilibrium brain tissue magnetization and TIb = 1650 ms the decay time for labeled blood at 3 Tesla [50]. For improvement of SNR, we calculated average CBF for the time-series. We then coregistered averaged CBF maps to T1-weighted UNI volumes and extracted regional CBF using binary masks derived from *DL* + *DiReCT* (individual space, AsegAtlas). Regional CBF of bilateral hippocampi and amygdalae and anterior/posterior hippocampi were normalized for global mean CBF within gray matter. We calculated framewise displacement using ASL realignment parameters to sum the absolute movement values of the differentiated realignment estimates from one volume to the next. For statistical comparison, we calculated mean framewise displacement (FD) of the whole time series for each subject and scan session [51].

### Statistics

Based on previous studies [4, 52], we expected to get a moderate effect size of f^2^ = 0.15 for the between factor (ECT, TAU-responders, HC) having a total sample size of 60 participants and performing a mixed-model MANCOVA with one between factor, five dependent variables, two within factors, and controlling for four covariates. A post-hoc analysis to calculate the power achieved, given alpha = 0.05, sample size = 60, number of groups = 3, number of dependent variables = 5, and number of predictors = 6, yielded a power (1-beta) of 0.98 using the program G*Power [53].

We used the Statistical Package for Social Sciences SPSS 27.0 (SPSS, Inc., Chicago, Illinois) for data analyses. Dimensional demographic variables between groups were compared using two-sample t-tests or ANOVAs as appropriate. Categorical variables were compared using χ^2^ tests (see Table 1). To investigate differences in neuroplasticity between groups, we compared time effects between the two MRI-scans for patient groups and HC. Continuous variables were tested for normal distribution and homoscedasticity using Kolmogorov Smirnov and Levene’s tests. A repeated measures MANCOVA with the independent variable group (ECT, TAU-responders, HC), the dependent variables volume hippocampus, volume amygdala, CBF hippocampus, CBF amygdala, and global mean cortical thickness, the within subject factors timepoint and hemisphere and the covariates age, sex and motion parameters (mean-FD-baseline and mean-FD-difference (follow up – baseline)) was calculated. In case of a significant group × time main effect post hoc ANCOVAs of group × time effects were considered for each region and imaging modality using the same within subject factors and covariates. Effect sizes for the MANCOVA and post hoc ANCOVAS were reported using η^2^ [54].

To explore which groups drive significant group × time effects of post hoc ANCOVAs significant results were followed up with paired *t*-tests comparing baseline with follow up measures of the respective neuroimaging variables within the three groups. In case of significant results of paired *t*-tests for volume and CBF in the hippocampi, we additionally calculated exploratory follow up group comparisons for the anterior and the posterior hippocampi [5, 23]. Significant results of paired t-tests investigating volumetric changes in the hippocampi and the amygdalae were complemented using *FreeSurfer* to investigate changes in volume in hippocampal subfields and subnuclei of the amygdalae. Significant results of paired *t*-tests for global mean cortical thickness were followed up with exploratory paired *t*-tests for regions that were associated with ECT-treatment response in previous studies [19, 24, 25]: insula, caudal ACC, medial OFC and lateral OFC. Effect sizes for post hoc *t*-tests were reported using Cohen’s d.

For all follow up paired t-tests with significant results, we calculated exploratory correlations between the difference of the imaging variable (follow up - baseline) and the overall clinical improvement (HAMD follow up - baseline) in the respective groups (ECT or TAU-responder). Furthermore, we calculated exploratory correlations between number of ECT-sessions and those imaging modalities that differed between timepoints (see Supplementary Material, S5). All tests were two-tailed and a level of significance of *p* < 0.05 was applied.

## Results

ECT, TAU-responders, and HC did not differ regarding age, sex, handedness, and time between scans (see Table 1). ECT and TAU-responders did not differ regarding depression severity at baseline. ECT-patients had a longer duration of the current episode and a higher number of total episodes (see Table 1). Seven patients in the ECT-group and two TAU-responders met criteria for double-depression for many years (mean duration ECT-group: 15.1 ± 8 years, mean duration TAU-responders: 18.5 ± 12 years).

### Group comparisons

**Fig. 2:**
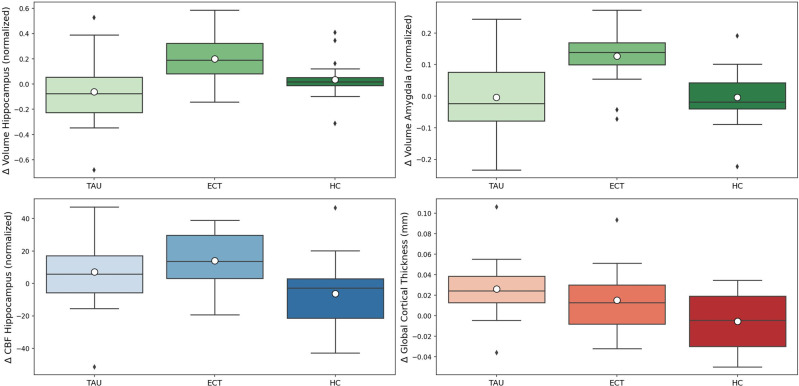
Group differences between timepoints (follow up – baseline) in subcortical and cortical MRI measurements. CBF cerebral blood flow, ECT electroconvulsive therapy group, HC healthy controls group, TAU treatment as usual group (TAU-responders).

The MANCOVA revealed a significant group × time interaction main effect showing a large effect size (F (10, 98) = 5.384, *p* < 0.001, η^2^ = 0.355), but no significant hemisphere × group × time interaction (F (10, 98) = 1.263, *p* = 0.262, η^2^ = 0.114). Post hoc ANCOVAs showed a significant group × time interaction for hippocampal volume (F (2, 53) = 8.005, *p* < 0.001, η^2^ = 0.232), amygdalar volume (F (2, 53) = 9.771, *p* < 0.001, η^2^ = 0.269), CBF of the hippocampi (F (2, 53) = 5.115, *p* = 0.009, η^2^ = 0.162), and global mean cortical thickness (F (2, 53) = 9.148, *p* < 0.001, η^2^ = 0.257) but not for CBF of the amygdalae (F (2, 53) = 2.123, *p* < 0.130, η^2^ = 0.074). ANCOVAs of measurements (averaged across both hemispheres) with a significant group × time interaction are visualized as boxplots (see Fig. 2).

Significant group × time interactions were driven by volume increase in bilateral hippocampi, bilateral amygdalae, and CBF increase in bilateral hippocampi in the ECT-group. Global mean cortical thickness showed an increase in bilateral hemispheres in TAU-responders, and in the right hemisphere in the ECT-group (see Table 2).

**Table 2:** Follow up paired *t*-tests for post hoc ANCOVAs with significant group × time interaction.

Region of interest	ECT-group	TAU-responders	HC
Volume hippocampus (l)	T(19) = −3.1, ***p*** = **0.006**, d = −0.70	T(19) = 1.1, *p* = 0.28, d = 0.25	T(19) = −1.7, *p* = 0.11 d = −0.38
Volume hippocampus (r)	T(19) = −6.4, ***p*** < **0.001**, d = −1.4	T(19) = 0.8, *p* = 0.43 d = 0.18	T(19) = 0.1, *p* = 0.91 d = 0.026
Volume amygdala (l)	T(19) = −4.6, ***p*** < **0.001**, d = −1.0	T(19) = 0.3, *p* = 0.80 d = 0.06	T(19)=−0.07, *p* = 0.94 d = −0.02
Volume amygdala (r)	T(19) = −7.3, ***p*** < **0.001**, d = −1.6	T(19) = −0.0, *p* = 0.99 d = −0.002	T(19) = 0.3, *p* = 0.76 d = 0.07
CBF hippo (l)	T(19) = −2.7, ***p*** = **0.014**, d = −0.60	T(19) = −1.5, *p* = 0.14 d = −0.35	T(19) = 1.5, *p* = 0.14 d = 0.34
CBF hippo (r)	T(19) = −3.4, ***p*** = **0.003**, d = −0.80	T(19) = −1.0, *p* = 0.34 d = −0.22	T(19) = 0.9, *p* = 0.39 d = 0.20
Mean cortical thickness (l)	T(19) = −1.5, *p* = 0.148, d = −0.34	T(19) = −3.3, ***p*** = **0.004**, d = −0.74	T(19) = -, *p* = 0.21, d = 0.29
Mean cortical thickness (r)	T(19) = −2.6, ***p*** = **0.018**, d = −0.58	T(19) = −4.3, ***p*** < **0.001**, d = −0.96	T(19) = -, *p* = 0.63, d = 0.11

*ECT* electroconvulsive therapy, *TAU* treatment as usual, *HC* healthy controls, *CBF* cerebral blood flow.

*P*-values marked with bold indicate statistically significant differences between groups (*p* < 0.05).

Exploratory follow up tests for volume and CBF in the anterior and posterior hippocampi in the ECT-group identified volume increase in bilateral anterior and posterior hippocampi and CBF-increase in bilateral anterior but not in posterior hippocampi (see Supplementary Material, S6). Analysis of changes in hippocampal subfields and subnuclei of the amygdalae revealed volume increase in 25 out of 44 hippocampal subfields and in 13 out of 20 amygdalar subnuclei (see Supplementary Material, S7). Exploratory follow up tests for cortical thickness in the subregions insula, caudal ACC, medial OFC and lateral OFC showed a spatial overlap between the ECT-group and TAU-responders in the right hemisphere; more specifically in the right insula and in the right lateral OFC (see Supplementary Material, S8–S10).

Exploratory correlations showed a positive correlation between symptom reduction in HAMD-total scores (follow up - baseline) and volume (in mm^3^) increase of the right posterior hippocampus in the ECT-group (see Supplementary Material, S11). Further correlations did not yield significant results.

## Discussion

This is the first study that directly compares longitudinal assessments of subcortical volumes and CBF (amygdala and hippocampus) and cortical thickness between an ECT-group, TAU-responders and HC. Global mean cortical thickness and cortical thickness in the right insula and in the right lateral OFC increased in both ECT and in TAU-responders pointing to a potential role of increase in cortical thickness for treatment response. In contrast, volume increase in the hippocampi and the amygdalae and CBF increase in the hippocampi were only observed in the ECT-group and occurred neither in TAU-responders nor in HC. CBF in the amygdala did not change over time.

The clinical importance of ECT-induced structural and functional brain changes is largely unresolved. Therefore, it seems critical to investigate which ECT-induced brain changes are associated with treatment response. Assuming that ECT-induced changes are responsible for clinical improvements such changes should also occur in patients with depression that respond to other treatment modalities (e.g. antidepressive medication and psychotherapy). Indeed, we found an increase in global mean cortical thickness in both the ECT-group and in TAU-responders. Follow up tests yielded large effect sizes for increase in cortical thickness in bilateral insulae in the ECT-group (d = 1.2 for the left hemisphere and d = 1.5 for the right hemisphere) and in TAU-responders (d = 0.8 for the right hemisphere). Furthermore, there was an overlap of local increase in cortical thickness in the right lateral OFC in both groups while an increase in cortical thickness in bilateral caudal ACC was only observed in the ECT-group (see Supplementary Tables S8 and S9). ECT-induced increase in cortical thickness in the insula and the lateral OFC have been linked to treatment outcome before [19, 24, 25]. Furthermore, local increase in cortical thickness in the OFC and the ACC were reported in MDD treatment responders to antidepressive medication [28, 30]. This is the first study that statistically compares alterations of cortical thickness between an ECT-treatment group and TAU-responders. The similarity regarding the localization of increase in cortical thickness is striking and represents a line of evidence complementary to correlational analysis suggesting that increase in cortical thickness may be a mechanism of response plasticity in depression.

The neuronal basis of such increase in cortical thickness cannot be assessed directly based on MRI-measures. Given that neuropil (unmyelinated axons, dendrites and glial cell) has a greater impact on cortical thickness than cell bodies [55] it has been argued that increase in cortical thickness is primarily driven by axonal branching, synaptogenesis and glia cells [24, 25]. This may be a consequence of ECT-induced increase in brain-derived neurotrophic factor [56], which precedes glia and angiogenesis [57]. While high-field MRI provides an increased resolution that may advance our understanding regarding cortical layers [58] histological studies would be required to provide definitive answers on the biological basis of increase in cortical thickness related to ECT-treatment and response plasticity.

Our finding of ECT-induced volume increase in the hippocampi and the amygdalae are well in line with prior studies [7, 10]. However, the clinical relevance of those volume increases are a matter of debate [4, 7]. A previous study found that ECT-treatment response is associated with CBF increase in the posterior hippocampus and volume increase in the anterior hippocampus [23]. We found a volume increase in both anterior and posterior hippocampi. Exploratory correlational analyses of our analyses suggest that volume increase in the right posterior hippocampus is associated with a decrease in overall depression severity (see Supplementary Material, S11). CBF increase was observed in the anterior but not in the posterior hippocampi (see Supplementary Material, S6), however alterations in CBF were not associated with clinical improvements. Thus, our finding adds to a rather complex picture regarding findings specific for sub-compartments or hippocampal subfields that clearly need further replication [7, 22, 23].

We did not find any significant changes of volume or CBF in the hippocampi and amygdalae in TAU-responders. A large longitudinal study investigating hippocampal volumes in patients with MDD found that successful antidepressive treatment prevents the hippocampus from further volume decline, but does not lead to volume increase [27]. This is consistent with our finding of unchanged volumes in TAU-responders. Thus, it is possible that ECT induces structural and functional changes in hippocampal brain regions that accelerate, and exceed the neurobiological underpinnings of response plasticity in patients receiving TAU. Future studies should use high-field MRI to specifically investigate hippocampal subregions. This may contribute to elucidate the clinical relevance of ECT-induced hippocampal changes [22].

This study has some limitations. First, limitations of observational study apply: there is a risk for systematic biases between patient groups and no causal inferences can be drawn. Second, patient groups are inhomogeneous regarding sex, chronicity (duration and number of episodes), and diagnoses (the ECT-group also includes depressed patients with bipolar disorder). Third, patients were not required to be on stable medication and medication was changed during study participation. Fourth, we compare a group of ECT-patients (regardless of treatment outcome) with TAU-responders. This approach enables to identify shared neuroplastic changes that may underlie clinical improvement. Larger samples could extend our findings and enhance specificity by comparing ECT-responders/ non-responders with TAU-responders/non-responders. Fifth, the study is underpowered to perform robust correlational analyses with clinical rating scales and correlational analyses are exploratory in nature [59].

In sum, this is the first study to use multimodal MRI to compare ECT-induced structural and functional changes with the neurobiology of TAU-responders. This design allows for putting the ECT effects into perspective. Our results suggest that ECT-induced changes of increase in cortical thickness mimic response plasticity to TAU in MDD, particularly in the right insula and the right lateral OFC. This suggests that increase in cortical thickness may play a role for the process of treatment response in depression. Future studies should reinvestigate the clinical importance of changes of perfusion and volume in the hippocampi and the amygdalae using larger samples that allow to account for clinical variability during the ECT-treatment course [4].

## Supplementary information

Supplementary material
