# Supplementary material for: Distinct and shared patterns of brain plasticity during electroconvulsive therapy and treatment as usual in depression: an observational multimodal MRI-study
Source: Transl Psychiatry. 2023 Jan 10;13:6. doi: 10.1038/s41398-022-02304-2 (PMC9832014; doi:10.1038/s41398-022-02304-2)

Supplementary material S1: Study flow chart
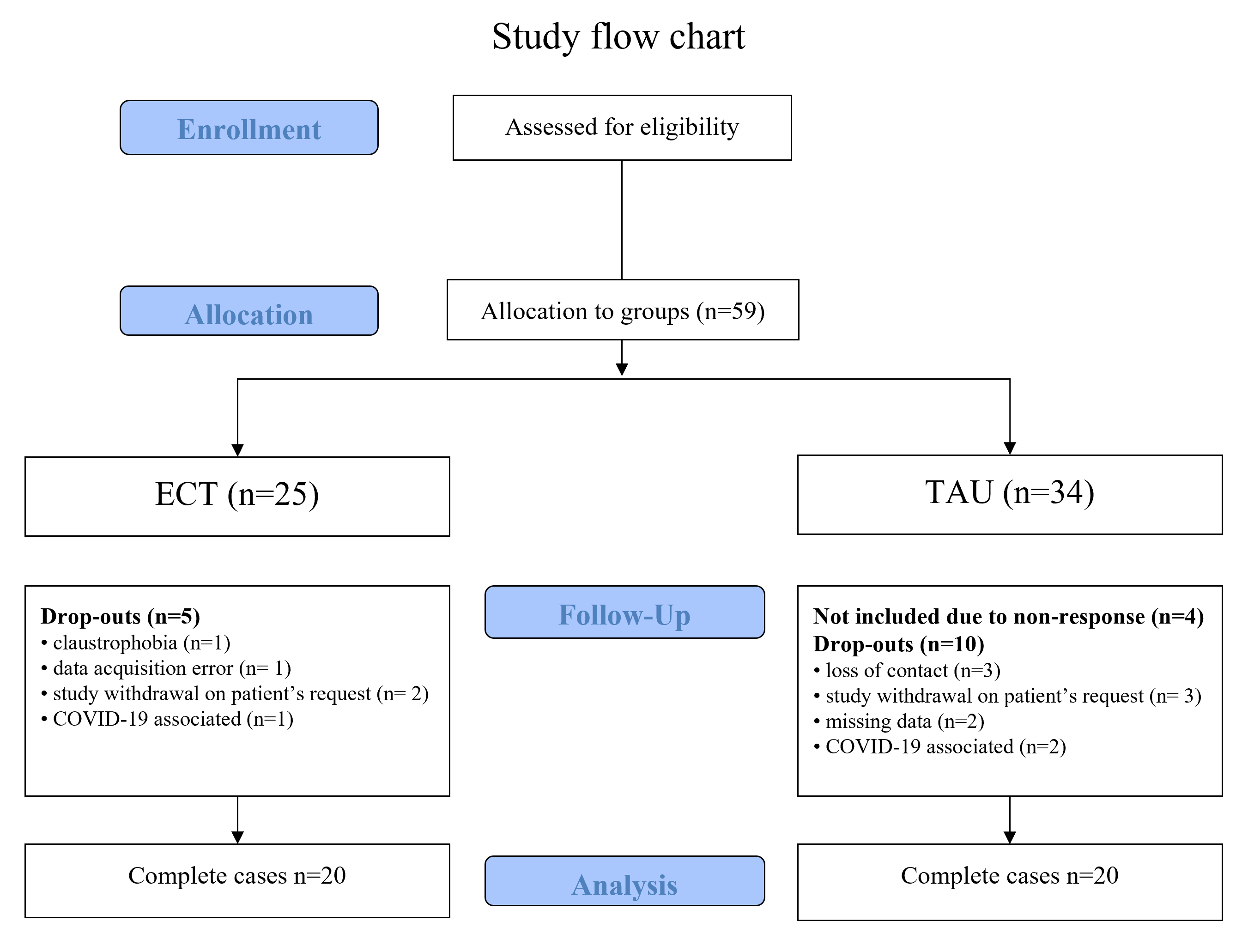


Patients were assessed for eligibility during daily clinical routine. Allocation to study groups of this observational study was made according to administered treatment (ECT, electroconvulsive therapy or TAU, treatment as usual).

Supplementary material S2: Paired t-test between 1^st^ and 2^nd^ MRI-scan for TAU non-responders (n=4)

| **Demographics TAU non-responders** |  |
| --- | --- |
| Age | 43.25 ± 16 |
| Sex | female n=1, male= 3 |
| Days between MRI-scans | 95±16 |
| HAMD-total (1^st^ MRI-scan) | 21.8 ± 7 |
| HAMD-total (2^nd^ MRI-scan) | 18.3 ± 6 |
| **Region of interest** | **Statistics** |
| Volume hippocampus (left) | T(3)=0.4, p=0.74 |
| Volume hippocampus (right) | T(3)=1.6, p=0.22 |
| Volume amygdala (left) | T(3)=2.5, p=0.08 |
| Volume amygdala (right) | T(3)=1.4, p=0.27 |
| CBF hippo (left) | T(3)=1.4, p=0.27 |
| CBF hippo (right) | T(3)=-0.8., p=0.52 |
| CBF amygdala (left) | T(3)=-1.2., p=0.33 |
| CBF amygdala (right) | T(3)=0.6., p=0.58 |
| Mean cortical thickness (left) | T(3)=0.6, p=0.60 |
| Mean cortical thickness (right) | T(3)=1.0, p=0.41 |

Abbreviations: HAMD: Hamilton depression rating scale; TAU: treatment as usual; CBF: cerebral blood flow.

Supplementary material S3: Pre-processing of MRI data with FreeSurfer

First, we used *HD-BET* (<https://github.com/MIC-DKFZ/HD-BET>) for highly accurate brain extraction using the INV1 volumes and applied the derived binary mask to the UNI volumes. We then reoriented MRI data from baseline acquisition to MNI orientation and coregistered MRI data from follow-up to the corresponding baseline data using *SPM12* (<https://www.fil.ion.ucl.ac.uk/spm/>). Coregistration derived non-zero values outside the brain were reset to zero using the image calculation function of *SPM12*. Next, we performed cortical reconstruction and volumetric segmentation of MRI scans including segmentation of hippocampal subfields and amygdalar nuclei using standard settings in *FreeSurfer* 7.0 (<http://surfer.nmr.mgh.harvard.edu/>). Finally, we extracted cortical thickness and subcortical volumes using *asegstats2table* and Python scripting. All calculations were performed on UBELIX (<http://www.id.unibe.ch/hpc>), the HPC cluster at the University of Bern.

Supplementary material S4: Comparison between *FreeSurfer* and *DL+DiReCT*

*
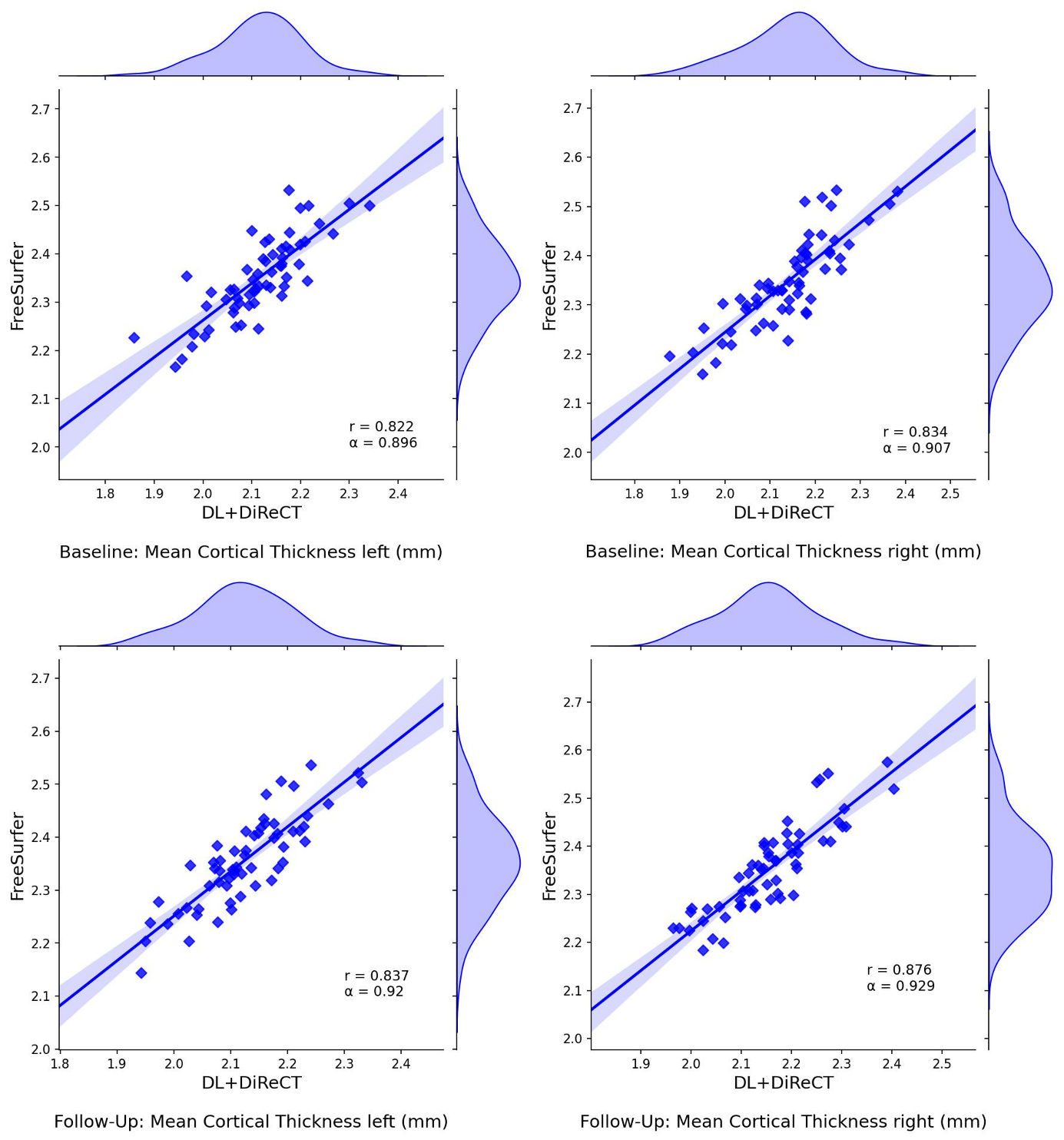
*

Pearson correlations and Cronbach’s α are shown for measures of cortical thickness derived from *FreeSurfer* and *DL+DiReCT*


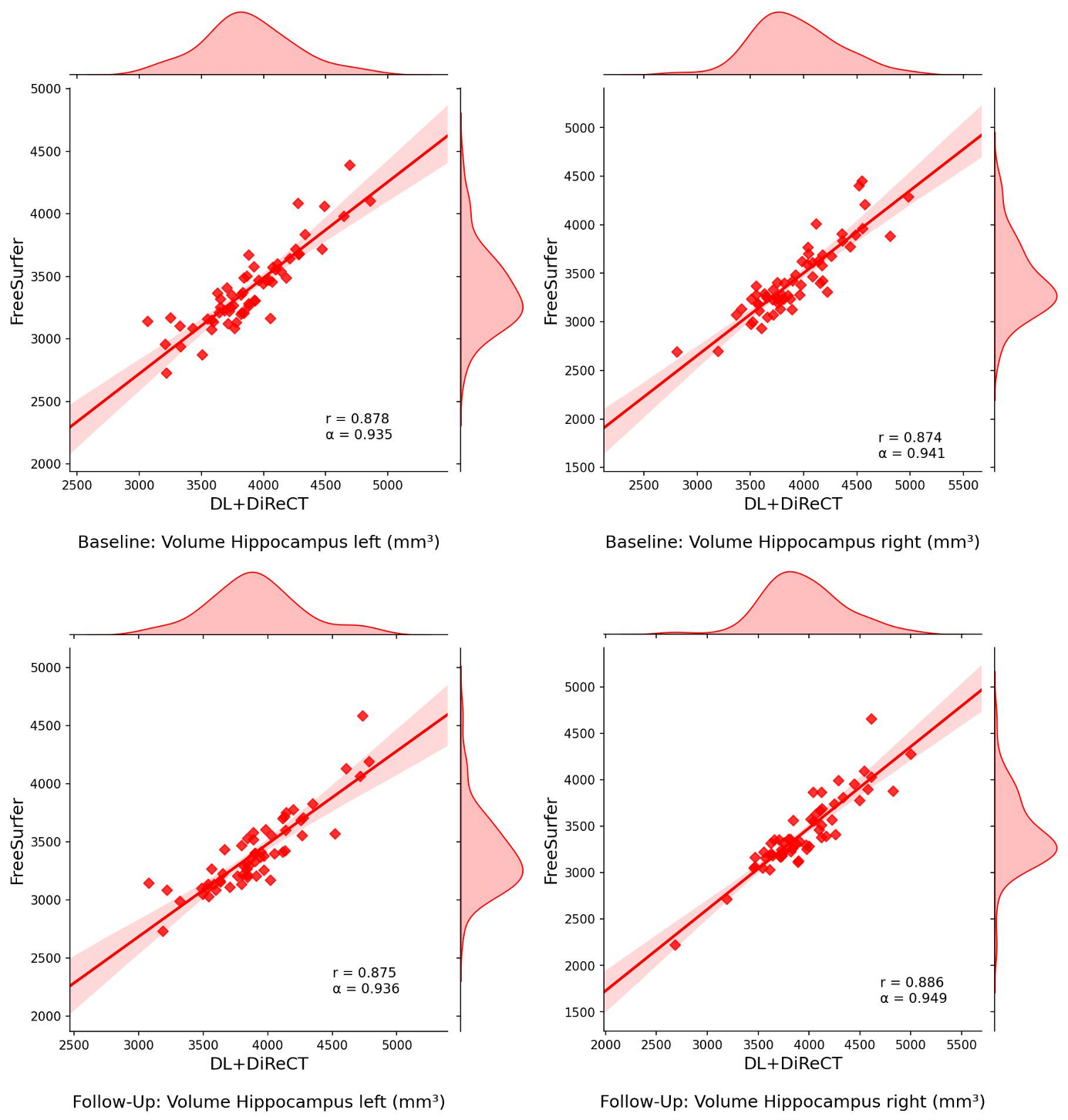


Pearson correlations and Cronbach’s α are shown for estimates of hippocampal volumes derived from *FreeSurfer* and *DL+DiReCT*

*
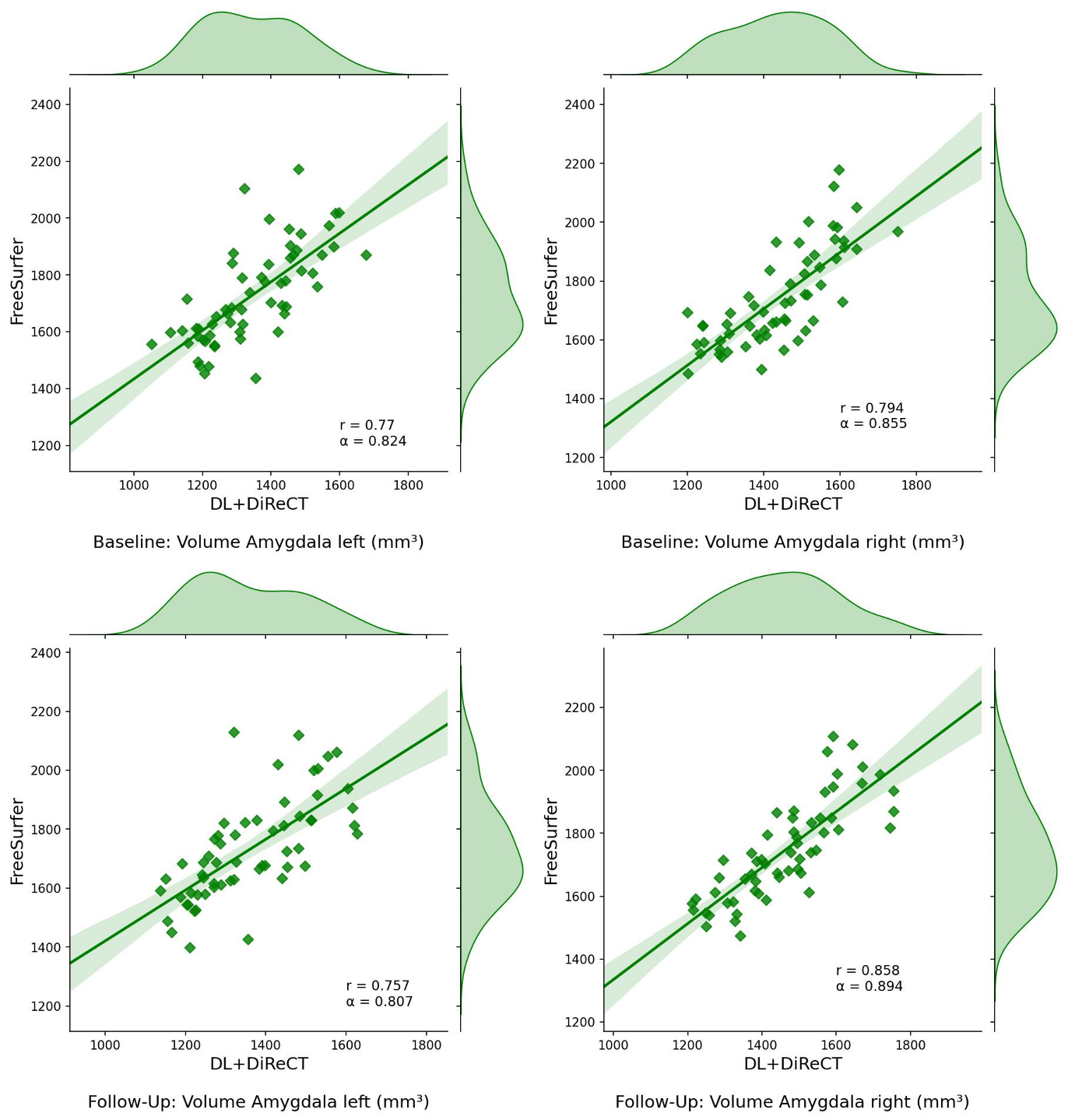
*

Pearson correlations and Cronbach’s α are shown for estimates of amygdalar volumes derived from *FreeSurfer* and *DL+DiReCT*

Supplementary material, S5: Spearman’s correlations between difference of imaging variables (follow up - baseline) with significant longitudinal changes and number of ECT sessions

|  | Vol HL | Vol HR | Vol AL | Vol AR | CBF HL | CBF HR | CT R |
| --- | --- | --- | --- | --- | --- | --- | --- |
| ECT | r=0.331  p=0.16 | r=0.195  p=0.41 | r=-0.15  p=0.95 | r=-0.004  p=0.99 | r=-0.094  p=0.70 | r=-0.119  p=0.62 | r=-0.149  p=0.53 |
| Vol HL |  | **r=0.495**  **p=0.03** | r=0.173  p=0.47 | r=0.368  p=0.11 | r=0.039  p=0.87 | r=0.122  p=0.61 | **r=0.456**  **p=0.04** |
| Vol HR |  |  | r=-0.152  p=0.53 | r=0.035  p=0.89 | r=0.117  p=0.63 | r=0.087  p=0.71 | r=0.224  p=0.34 |
| Vol AL |  |  |  | **r=0.564**  **p=0.10** | r=-0.167  p=0.48 | r=-0.173  p=0.47 | r=0.334  p=0.15 |
| Vol AR |  |  |  |  | r=-0.200  p=0.40 | r=-0.120  p=0.61 | r=0.442  p=0.05 |
| CBF HL |  |  |  |  |  | **r=0.91**  **p<0.001** | r=-0.302  p=0.20 |
| CBF HR |  |  |  |  |  |  | r=-0.180  p=0.45 |

Abbreviations: ECT: number of ECTs, Vol: Volume; HL: hippocampus left; HR: hippocampus right; AL: amygdala left; AR: amygdala right; CBF: cerebral blood flow; CT R: mean global cortical thickness right

Supplementary material, S6: Paired t-tests within the ECT group (baseline vs. follow up)

| Region of interest | Left hippocampus | Right hippocampus |
| --- | --- | --- |
| Volume anterior hippocampus | T(19)=-2.6, **p=0.018**, d=-0.58 | T(19)=-4.4 , **p< 0.001**, d=-1.0 |
| Volume posterior hippocampus | T(19)= -2.5, **p=0.020**, d=-0.57 | T(19)=-4.1 , **p<0.001**, d=-0.92 |
| CBF anterior hippocampus | T(19)=-2.5 , **p=0.024**, d=-0.55 | T(19)= -2.8, **p=0.011**, d=-0.63 |
| CBF posterior hippocampus | T(19)=-0.4, p=0.673, d=-0.10 | T(19)=-1.9, p=0.062, d=-0.44 |

Supplementary material S7: Analysis of hippocampal subfields and subnuclei of the amygdalae in the ECT-group using *FreeSurfer*


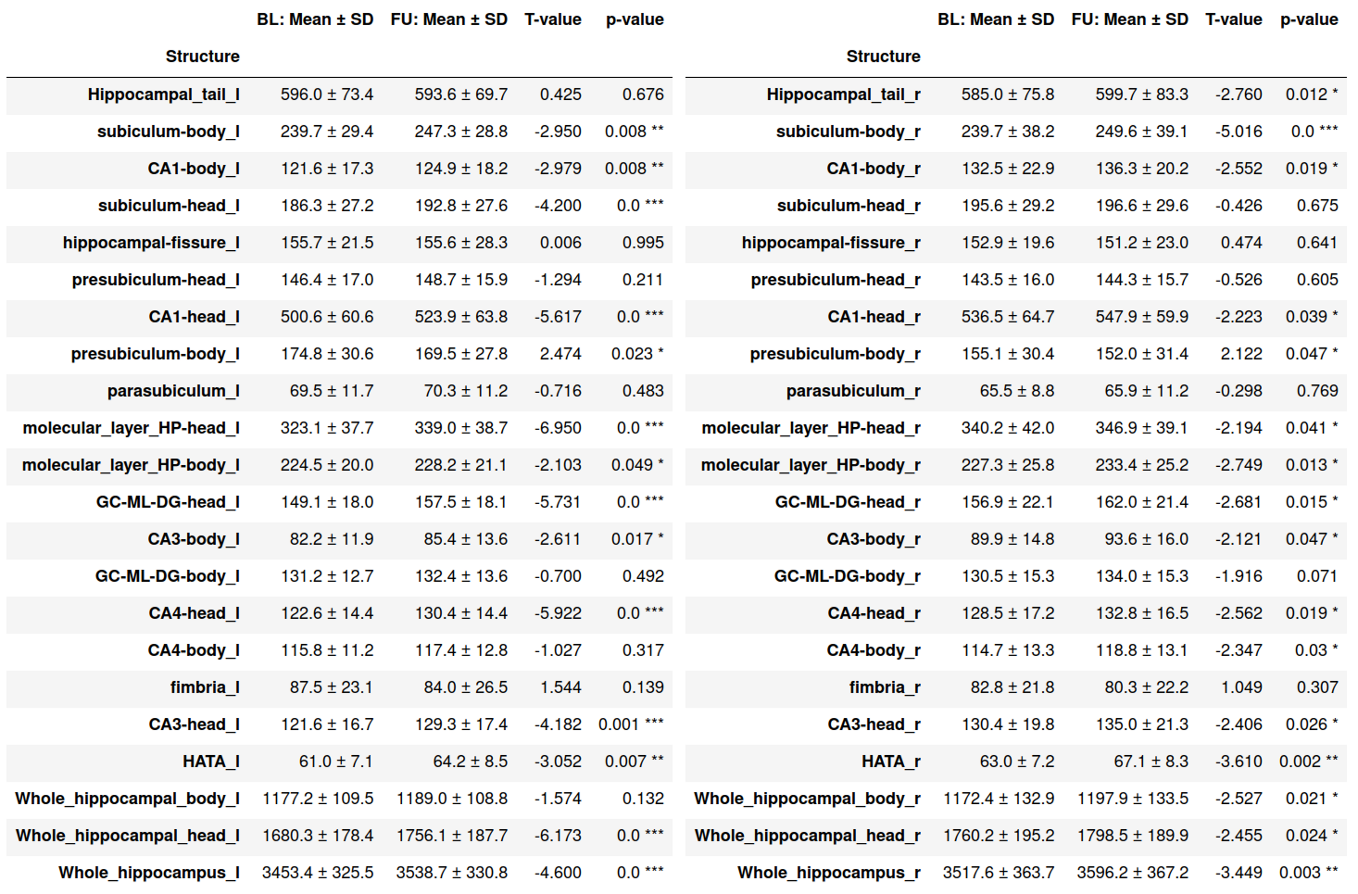


Volume increases (Follow up (FU) > Baseline (BL) are found in 30 out of 44 hippocampal subfields using a two tailed level of significance of p<0.05.


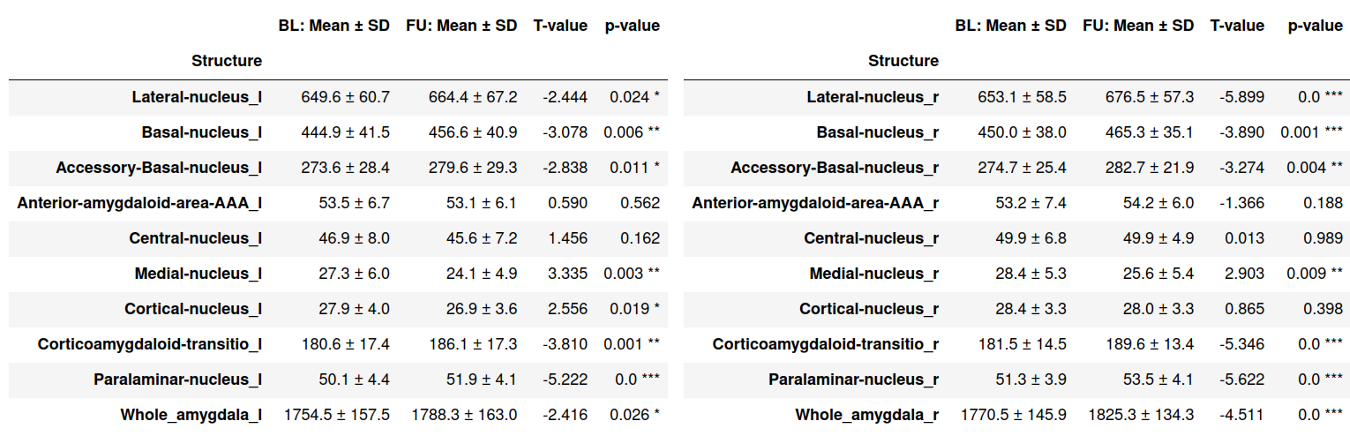


Volume increases (Follow up (FU) > Baseline (BL) are found in 15 out of 20 amygdalar subnuclei using a two tailed level of significance of p<0.05.

Supplementary material, S8: Paired t-tests within the ECT-group (baseline vs. follow up)

| Thickness | Left hemisphere | Right hemisphere |
| --- | --- | --- |
| Insula | T(19)= -5.3, **p<0.001**, d=-1.17 | T(19)= -6.5, **p<0.001**, d=-1.46 |
| Caudal ACC | T(19)= -3.6, **p=0.002**, d=-0.79 | T(19)= -2.4, **p=0.026**, d=-0.54 |
| Medial OFC | T(19)=0.43 , p=0.673, d=0.10 | T(19)= -1.6, p=0.122, d=-0.36 |
| Lateral OFC | T(19)= 0.60, p=0.560, d=0.13 | T(19)=-2.4 , **p=0.028**, d=-0.53 |

Supplementary material, S9: Paired t-tests within the TAU-responders (baseline vs. follow up)

| Thickness | Left hemisphere | Right hemisphere |
| --- | --- | --- |
| Insula | T(19)= -0.9, p=0.372, d=-0.20 | T(19)=-3.7, **p=0.002**, d=-0.83 |
| Caudal ACC | T(19)= 0.2, p=0.868, d=-0.04 | T(19)= -1.6, p=0.129, d=-0.36 |
| Medial OFC | T(19)=-0.4 , p=0.709, d=-0.09 | T(19)=-3.0, **p=0.008**, d=-0.66 |
| Lateral OFC | T(19)=-0.7 , p=0.483, d=-0.16 | T(19)=-2.4 , **p=0.026**, d=-0.54 |

Supplementary material, S10: Paired t-tests within HC (baseline vs. follow up)

| Thickness | Left hemisphere | Right hemisphere |
| --- | --- | --- |
| Insula | T(19)= 1.69, p=0.107, d=0.38 | T(19)= 0.09, p=0.930, d=0.02 |
| Caudal ACC | T(19)= 0.72, p=0.483 d=0.16 | T(19)=0.26, p=0.795, d=0.06 |
| Medial OFC | T(19)= 0.74, p=0.471, d=0.16 | T(19)= -0.80, p=0.439, d=-0.18 |
| Lateral OFC | T(19)= -1.2, p=0.247, d=-0.27 | T(19)=-1.1 , p=0.294, d=-0.24 |

Supplementary material, S11: Spearman correlation (r= -0.494*, p=0.027) between difference HAMD (follow up - baseline) and volume of the right posterior hippocampus in the ECT group.


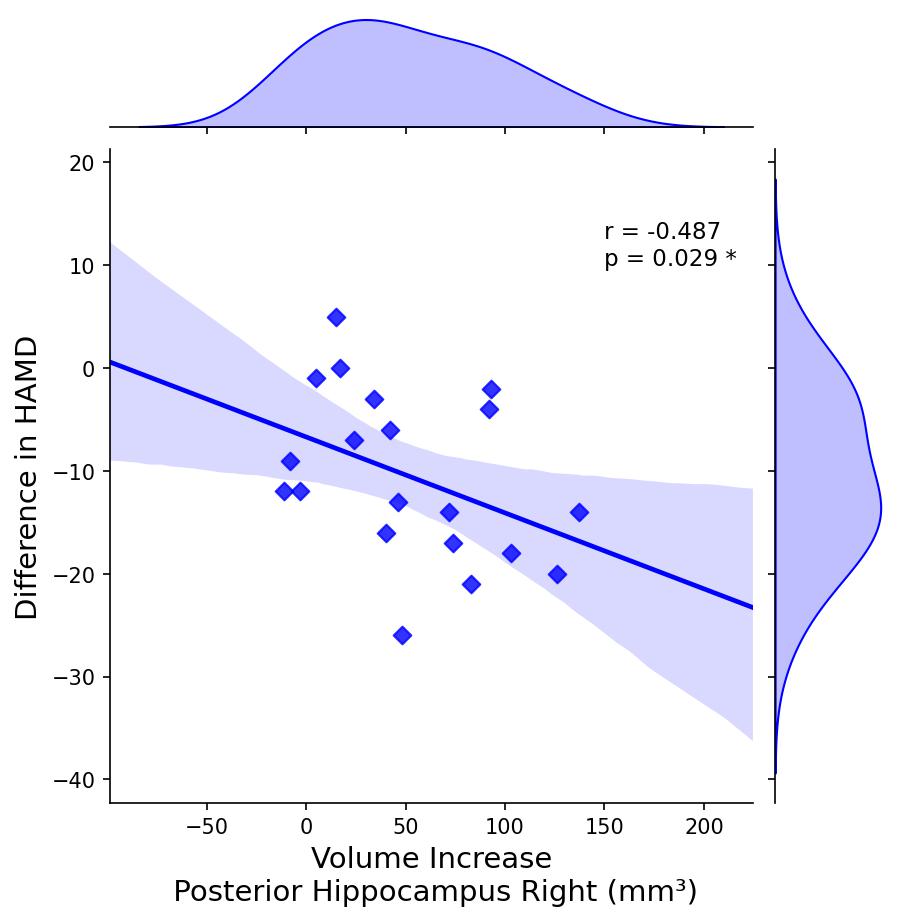

Supplement: Supplementary file 1 — Supplementary material [file 41398_2022_2304_MOESM1_ESM.docx]
